# Memoirs and Letters of Sir James Paget

**Published:** 1902-09

**Authors:** 


### Reviews of Books.

**Memoirs and Letters of Sir James Paget.** Edited by STEPHEN PAGET, one of his sons. Second Edition. Pp. 438. London: Longmans, Green and Co.

This book is founded to a great extent on some records of his life written by Sir James Paget himself, with a commentary on these memoirs, and an account of his later years written by his son. To these are added numerous letters, chiefly to members of his family, which are inserted in chronological order, and give a very good idea of his thoughts on many subjects. Reproductions of portraits and photographs, especially a good photogravure plate of the famous picture of Paget by Sir John Millais, enhance the value of the book.

The actual autobiography is in many respects the most interesting, possessing the charm and clear literary style of all Sir James's writings, and giving a vivid picture of his boyhood and the struggles of his youth and early manhood. What a life of high aims and strenuous endeavour he depicts! Through poverty, disappointment, deferred hope, to the well-earned reward of success and fame!

One is much struck with the immense capacity for work, and of work requiring untiring patience and hope, Sir James Paget must have possessed, and one realises how impossible such a career would be without splendid physical as well as mental endowments. These he inherited from both his parents. "I can boast," he says, "of being, in the best sense, well-born." From his father he obtained neatness, punctuality, and business habits; from his mother "activity and industry, and admiration for all that was beautiful in art and nature."

The story of his life is told in simple unpretentious language, and some attempt is made to really portray the man himself; but although we learn a great deal about Sir James Paget, it cannot be said that this is a biography of the very best sort—that sort, namely, which by many graphic touches and minute description of dress, manners, conversation, &c., gives us a living picture which we learn to know. But it is, nevertheless, well written and well compiled, and we are enabled to follow with admiration the onward progress of a great man. One cannot, indeed, sum up his life better than by quoting the last sentence of the first part of this book: "But all his life he moved upward; from the lower level of the early years, and the limited range of vision, to the time when he attained success in practice, and could see all round him; then, higher still, having left success behind him, in the increasing loneliness of old age; and last of all, in the time of utmost infirmity, highest of all."

